# Current Status of Treatment of Spinal and Bulbar Muscular Atrophy

**DOI:** 10.1155/2012/369284

**Published:** 2012-06-07

**Authors:** Fumiaki Tanaka, Masahisa Katsuno, Haruhiko Banno, Keisuke Suzuki, Hiroaki Adachi, Gen Sobue

**Affiliations:** Department of Neurology, Nagoya University Graduate School of Medicine, 65 Tsurumai-cho, Showa-ku, Nagoya 466-8550, Japan

## Abstract

Spinal and bulbar muscular atrophy (SBMA) is the first member identified among polyglutamine diseases characterized by slowly progressive muscle weakness and atrophy of the bulbar, facial, and limb muscles pathologically associated with motor neuron loss in the spinal cord and brainstem. Androgen receptor (AR), a disease-causing protein of SBMA, is a well-characterized ligand-activated transcription factor, and androgen binding induces nuclear translocation, conformational change and recruitment of coregulators for transactivation of AR target genes. Some therapeutic strategies for SBMA are based on these native functions of AR. Since ligand-induced nuclear translocation of mutant AR has been shown to be a critical step in motor neuron degeneration in SBMA, androgen deprivation therapies using leuprorelin and dutasteride have been developed and translated into clinical trials. Although the results of these trials are inconclusive, renewed clinical trials with more sophisticated design might prove the effectiveness of hormonal intervention in the near future. Furthermore, based on the normal function of AR, therapies targeted for conformational changes of AR including amino-terminal (N) and carboxy-terminal (C) (N/C) interaction and transcriptional coregulators might be promising. Other treatments targeted for mitochondrial function, ubiquitin-proteasome system (UPS), and autophagy could be applicable for all types of polyglutamine diseases.

## 1. Introduction

Spinal and bulbar muscular atrophy (SBMA) was first described in 1897 by a Japanese neurologist, Kawahara [1], and has been known worldwide as Kennedy's disease since 1968 when reported by Kennedy [2]. It is characterized by the degeneration and loss of lower motor neurons in the brainstem and spinal cord, and patients present with weakness and wasting of the facial, bulbar, and limb muscles, along with sensory disturbances and endocrinological abnormalities [3, 4]. SBMA is an X-linked trinucleotide polyglutamine disease, caused by an abnormal expansion of tandem CAG repeat in exon 1 of the androgen receptor (AR) gene on chromosome Xq11-12 [5]. In normal individuals, the CAG repeat ranges in size between 9 and 36, and expansion over 38 and up to 62 is pathogenic [5, 6]. Polyglutamine-expanded mutant AR accumulates in nuclei, undergoes fragmentation, and initiates degeneration and loss of motor neurons [7, 8].

 So far, nine polyglutamine diseases are known including SBMA, Huntington's disease, dentatorubral-pallidoluysian atrophy, and six forms of spinocerebellar ataxia (SCA), known as SCA1, SCA2, SCA3, SCA6, SCA7, and SCA17 [9, 10]. These diseases share several features such as late-onset, progressive neurodegeneration, anticipation, somatic mosaicism, and accumulation of misfolded mutant proteins in the nuclei or cytoplasm of neurons [8–13]. Expanded polyglutamine tracts form antiparallel beta-strands held together by hydrogen bonds formed between the main chain of one strand and the side chain of the adjacent strand. This leads the polyglutamine protein to acquire a nonnative beta-sheet conformation, which results in the accumulation of misfolded protein into microaggregates/oligomers and inclusions [3, 14]. Accumulation of polyglutamine-expanded protein into inclusions is considered to be protective [15–17], while diffuse nuclear microaggregates/oligomers might be toxic [18]. These aggregates and inclusions contain components of the ubiquitin proteasome system (UPS) and molecular chaperons, which attempt to degrade or refold the polyglutamine-expanded proteins [19]. Thus, these common features of aggregates and inclusions observed in polyglutamine diseases suggest that the expanded polyglutamine tract itself seems to be deeply involved in the pathogenesis.

**Figure 1:**
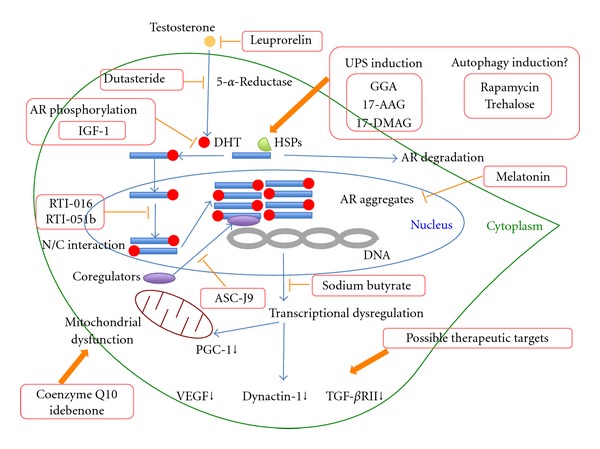
Potential disease-modifying therapies for spinal and bulbar muscular atrophy (SBMA). Ligand-induced nuclear translocation of mutant androgen receptor (AR) is a critical step of motor neuron degeneration in SBMA. In order to block this step, androgen deprivation therapies using leuprorelin and dutasteride have been developed. AR phosphorylation is another potential treatment strategy through attenuation of ligand binding. Insulin-like growth factor-1 (IGF-1) reduces mutant AR toxicity through phosphorylation of AR at the Akt consensus sites. Amino-terminal (N) and carboxy-terminal (C) (N/C) interaction of mutant AR is critical for toxicity, and this interaction is blocked by selective AR modulators such as RTI-016 and RTI-051b. The SBMA modifier melatonin blocks toxic fibrillar and induces nontoxic annular aggregates. As a transcription factor, the binding of AR to DNA in the nucleus is followed by the recruitment of a variety of transcriptional coregulators. 5-Hydroxy-1,7-bis(3,4-dimethoxyphenyl)-1,4,6-heptatrien-3-one (ASC-J9) disrupts the interaction between AR and its coregulators and yields a therapeutic effect. In SBMA, histone acetylation is impaired, resulting in transcriptional dysregulation. Sodium butyrate, histone deacetylase (HDAC) inhibitor is effective at this step. Furthermore, transcriptionally attenuated genes such as vascular endothelial growth factor (VEGF), dynactin-1, and transforming growth factor *β* receptor type II (TGF-*β*RII) are also possible therapeutic targets. Decreased expression of peroxisome proliferator-activated receptor *γ* coactivator 1 (PGC-1) is one of the causes of mitochondrial dysfunction, and treatments with the antioxidants coenzyme Q10 and idebenone have been developed targeting mitochondria. Mutant AR is degraded through induction of the ubiquitin-proteasome system (UPS) by acyclic isoprenoid geranylgeranylacetone (GGA) and heat shock protein 90 (Hsp90) inhibitors such as 17-(allylamino)-17-demethoxygeldanamycin (17-AAG) and 17-(dimethylaminoethylamino)-17-demethoxygeldanamycin (17-DMAG). Autophagy induction using rapamycin and trehalose is also effective for AR degradation in fly and cell models of SBMA. However, the opposite results concerning autophagy augmentation therapy were recently reported in SBMA knock-in mice.

 However, the observation that the same genetic mutation in nine different proteins results in nine different diseases highlights both the significance of a specific protein context other than the polyglutamine tract and the role of normal protein function in the pathogenesis of polyglutamine diseases [20]. Direct evidence that native protein functions and interactions may mediate toxicity comes from an animal model in which overexpression of wildtype AR harboring nonexpanded polyglutamine tract results in pathology resembling SBMA [21]. In the majority of polyglutamine diseases, neither the primary function nor the native interactors of the disease proteins are well known. SBMA represents an exception because AR protein structure and function as a ligand-dependent transcription factor are well characterized. AR belongs to the family of steroid hormone receptors and is composed of an amino-terminal domain, a DNA-binding domain, and a ligand-binding domain [22]. In the inactive state, AR is confined in the cytoplasm in association with heat shock proteins (HSPS). Testosterone binding to AR leads to the dissociation of AR from Hsps and causes nuclear translocation (Figure 1) [3, 23]. Also, ligand binding induces conformational changes of AR such as intra- or inter-molecular amino/carboxy-terminal (N/C) interactions (Figure 1) [3, 24]. Nuclear translocation of AR is followed by DNA binding to androgen-responsive elements, which in turn leads to recruitment of coregulators and expression regulation of androgen-responsive genes (Figure 1). These native functions and sequential processing of AR have important roles for the pathogenesis and therapy development of SBMA.

 In SBMA, expanded polyglutamine tracts are associated to lower levels of transcription of androgen-responsive genes [25, 26], which in turn lead to mild androgen insensitivity symptoms such as gynecomastia, feminized skin changes, testicular atrophy, and oligospermia/azoospermia causing reduced fertility [27]. However, dysregulation of androgen-responsive genes does not likely contribute to the neurological symptoms of SBMA, because complete androgen insensitivity syndrome associated with total loss of AR function has no signs of neurodegeneration [28], and AR knock out mice are also normal in motor neuron functions [29].

 So far, therapeutic interventions have been developed to target a number of events occurring through native AR functions upon ligand binding. Although no treatments have been established in SBMA, this review illustrates several therapeutic strategies based on the native function of AR and the common mechanisms shared by polyglutamine diseases.

## 2. Therapeutic Interventions to Inhibit Nuclear Transport of Mutant Androgen Receptor (AR)

Due to the X-linked transmission, SBMA exclusively affects males and is transmitted by clinically unaffected or mildly manifesting female carriers. A unique gender-specific feature of SBMA is well recapitulated in both vertebrate and invertebrate animal models of the disease [30, 31]. In transgenic mice expressing polyglutamine-expanded mutant AR, the disease fully manifests only in males due to higher levels of circulating androgens [30, 32, 33]. Importantly, decrease of androgen levels by castration of transgenic male mice prevents neurodegeneration, while treatment of transgenic female mice with testosterone induces disease manifestations [30]. In a fly model of SBMA, neurodegeneration occurs only if the flies are reared in a hormone-containing food [31], further supporting the ligand-dependent neurotoxicity of pathogenic AR. The prerequisite for SBMA pathogenesis is both the existence of ligand and nuclear translocation of mutant AR. This is shown by the observation that cytoplasmic retention of mutant AR by deletion of the nuclear localization signal suppresses polyglutamine-AR toxicity in SBMA mouse model [34].

 Leuprorelin is a potent luteinizing hormone-releasing hormone analog that decreases the production of testosterone and its more potent derivative, dihydrotestosterone (DHT), and has been used for the treatment of a variety of sex hormone-dependent diseases including prostate cancer, endometriosis, and central precocity [22]. Treatment of SBMA mice with leuprorelin reduced both polyglutamine-AR nuclear aggregation and inclusion formation in spinal cord as well as skeletal muscle and reversed the behavioral and histopathological phenotypes (Figure 1) [35]. These dramatic therapeutic effects of leuprorelin observed in a mouse model of SBMA were translated into a phase II clinical trial, and the patients treated with leuprorelin for 144 weeks exhibited significantly greater functional scores and better swallowing parameters than those who received a placebo [36]. Leuprorelin significantly diminished the serum level of creatine kinase and decreased mutant AR accumulation in scrotal skin of treated patients [36]. Of note, leuprorelin inhibited the nuclear accumulation and/or stabilization of mutant AR in the motor neurons of the spinal cord and brainstem of an autopsied patient who received it for 2 years [36]. More recently, a larger randomized placebo-controlled multicentric clinical trial of this drug showed no definite effect on motor functions, although swallowing function improved in a subgroup of patients whose disease duration was less than 10 years [37].

 Another potent drug for hormonal intervention is the 5-*α*-reductase inhibitor, dutasteride. The observation that motor neurons degenerating in SBMA express high levels of 5-*α*-reductase suggests that the conversion of testosterone to DHT represents a potential therapeutic target (Figure 1) [3]. However, the effectiveness of dutasteride was not proven in a 2-year double-blind placebo-controlled trial evaluated by the primary outcome measure of quantitative muscle assessment (QMA) [38].

 Although the results of these clinical trials are inconclusive, their findings do not exclude the possibility that ligand-targeted hormonal therapies slow the progression of SBMA. As an example of the effectiveness of longer administration of leuprorelin, a 75-year-old male SBMA patient who received leuprorelin for 5 years due to coexisting prostate cancer was reported to show long-term stabilization of motor function even when the treatment was started in the advanced stage of the disease [39].

 Besides the hormonal interventions, attenuation of ligand binding might be another therapeutic strategy for inhibition of nuclear transport of mutant AR. Ligand binding is at least partly mediated by phosphorylation of the mutant AR. Interestingly, substitution of the AR at two Akt consensus sites, S215 and S792, with aspartate, which mimics phosphorylation, reduces ligand binding, ligand-dependent nuclear translocation, transcriptional activation, and toxicity of expanded polyglutamine AR [40]. Furthermore, in motor neuron-derived MN-1 cells toxicity associated with polyglutamine-expanded, AR is rescued by coexpression with Akt [40]. Insulin-like growth factor-1 (IGF-1) reduces mutant AR toxicity in cultured cells through phosphorylation of AR at the Akt consensus sites [41]. Interestingly, augmentation of IGF-1/Akt signaling by overexpressing a muscle-specific isoform of IGF-1 selectively in skeletal muscle ameliorated the neurological phenotypes, extended the life span, and rescued not only muscle but also spinal cord pathology of SBMA transgenic mice [41]. This finding also indicates skeletal muscle as a viable target tissue for therapeutic intervention in SBMA. These results highlight that AR phosphorylation is another potential target for therapeutic intervention through inhibition of nuclear transport of mutant AR in SBMA (Figure 1).

## 3. Therapy Targeted for Conformational Changes of Androgen Receptor (AR) and Transcriptional Regulation

Ligand-mediated nuclear localization of the mutant AR is necessary but not sufficient for SBMA pathogenesis. Upon ligand binding, the AR undergoes several conformational changes including the interdomain interaction between the 23FQNLF27 motif near the amino terminus and the activation function-2 (AF2) domain near the ligand-bound carboxyl terminus (N/C interaction) [42, 43]. This N/C interaction is critical for toxicity through stabilizing the AR and enhancing hormone binding [44, 45]. Selective androgen receptor modulators such as RTI-016 and RTI-051b prevent the N/C interaction and ameliorated AR aggregation and toxicity while retaining AR transcriptional function, highlighting a novel therapeutic strategy for SBMA (Figure 1) [45].

 Another strategy for reducing toxicity of mutant AR is based on alteration of the morphology of the oligomers. Recently, Jochum and colleagues demonstrated that the pathogenic AR mutants formed oligomeric fibrils up to 300–600 nm in length, whereas annular oligomers 120–180 nm in diameter were formed by the nonpathogenic receptors [46]. They showed that melatonin ameliorated the pathological phenotype of the SBMA fly model through the conformational change of the polyglutamine-expanded oligomers from the toxic fibrillar forms to nontoxic annular forms (Figure 1) [46].

 As a ligand-dependent transcription factor, the binding of AR to DNA is followed by the recruitment of a variety of transcriptional coregulators, both coactivators and corepressors of transcription [47, 48]. In most steroid receptors, AF-2 domain plays a major role in receptor transactivation by serving as the interaction surface for transcriptional coregulators [3]. K720A and E897K mutations to the AF-2 attenuated polyglutamine-expanded AR toxicity in a *Drosophila* model of SBMA [20], suggesting that this toxicity requires DNA binding followed by association with coregulators through the AF-2 domain. In motor neurons, one of the key AR coregulators is the cAMP response element-binding (CREB)-binding protein (CBP), a transcriptional coactivator for neuronal survival factors [49]. In SBMA, through the sequestration of CBP by expanded polyglutamine aggregates [49, 50], many different transcription factors compete for functionally limiting levels of CBP, resulting in transcriptional disturbance. These observations raise the possibility of a therapeutic approach using compounds that disrupt the interaction of AR with transcriptional coregulators. One such compound is curcumin-related 5-hydroxy-1,7-bis(3,4-dimethoxyphenyl)-1,4,6-heptatrien-3-one (ASC-J9). ASC-J9 disrupts the interaction between AR and its coregulators including ARA70 and CBP (Figure 1) and markedly ameliorates phenotypes of SBMA transgenic mice by decreasing mutant AR aggregation [51]. ASC-J9 did not change the serum testosterone level in contrast to hormonal therapies associated with reduction of testosterone causing side effects on sexual functions.

 CBP exerts its transcriptional coactivating function through histone acetyltransferase (HAT) activity. Overexpression of CBP rescued histone acetylation and neurodegeneration in cell and animal models of SBMA [50, 52] in association with subsequent restoration of gene transcription, whereas histone deacetylase (HDAC) inhibitor also acetylates histone, suggesting that it may be of therapeutic value. Oral administration of sodium butyrate, an HDAC inhibitor, ameliorated neurological phenotypes as well as increased acetylation of nuclear histone in neural tissues of a mouse model of SBMA (Figure 1) [53]. Beneficial effects of this compound, however, were seen within a narrow therapeutic window of dosage.

 Downstream targets associated with decreased expression through transcriptional dysregulation in SBMA include vascular endothelial growth factor (VEGF), dynactin-1, and transforming growth factor *β* (TGF-*β*) receptor type II [54–57]. The importance of VEGF on maintenance of motor neuron is highlighted by motor neuron loss in mice with a homozygous deletion in the hypoxia-response element site in the VEGF promoter region [58]. Moreover, mutant AR-induced death of motor neuron-like cells (MN-1 cells) could be rescued by VEGF supplementation [54]. Dynactin-1 is a critical component of dynein/dynactin complex, a microtubule motor protein essential for retrograde axonal transport [59, 60], and its mutation was identified as the cause of a slowly progressive, autosomal dominant form of lower motor neuron disease [61]. In the mouse model of SBMA, pathogenic AR impairs retrograde axonal transport via transcriptional dysregulation of dynactin-1 [56]. TGF-*β* signaling was demonstrated to play a crucial role in the survival and function of adult neurons [62]. Transcriptional inhibition of TGF-*β* receptor type II suppressed nuclear translocation of phosphorylated Smad2/3, a key step in TGF-*β* signaling in the spinal motor neurons of SBMA mice and patients [57]. Targeting these molecules and as-yet-unknown transcriptionally dysregulated molecules might be another effective therapeutic approach (Figure 1).

## 4. Therapy Targeted for Ubiquitin-Proteasome System (UPS) and Autophagy System

The two major intracellular mechanisms for the degradation of misfolded proteins are the ubiquitin-proteasome system (UPS) and lysosome-mediated autophagy [23]. The degradation and removal of mutant AR may be obtained through overexpressing different Hsps, such as Hsp40 and Hsp70 through UPS pathway [63–65]. Moreover, the C-terminus of heat shock protein 70 interacting protein (CHIP) interacts with hsp90 or hsp70 and ubiquitylates misfolded proteins trapped by molecular chaperones and degrades them [19, 66]. Similar effects can also be induced by oral administration of the acyclic isoprenoid geranylgeranylacetone (GGA) [67]. GGA increased the levels of expression of Hsp70, Hsp90, and Hsp105, leading to inhibition of cell death, and amelioration of the neuromuscular phenotype of SBMA mice via activation of heat shock factor-1 and reduction of nuclear accumulation of polyglutamine-expanded AR (Figure 1) [67].

 In addition, Hsp90 inhibitors are able to promote the clearance of Hsp90 client proteins including misfolded mutant AR through the UPS. Treatment with potent Hsp90 inhibitors, such as 17-(allylamino)-17-demethoxygeldanamycin (17-AAG) or its derivative 17-(dimethylaminoethylamino)-17-demethoxygeldanamycin (17-DMAG), enhanced proteasomal degradation of the monomeric and aggregated mutant AR, reduced motor neuron degeneration, and increased survival in SBMA mice (Figure 1) [68–70]. It is to be noted that 17-AAG also disrupts the interaction between hsp90 and other client proteins, including steroid receptors, such as glucocorticoid receptor (GR), estrogen receptor-*α* (ER*α*), and retinoid X receptor-*α* (RXR*α*) [71], rendering them more susceptible to degradation and leading to unwanted side effects. Interestingly, a recent report shows that in motor neuron-derived cells 17-AAG removes misfolded AR species and aggregates by activating the autophagy system rather than UPS [72].

 This report indicates the importance of link and equilibrium between these two degradative systems [23]. In particular, HDAC6 plays an important role in the functional relationship between UPS and autophagy [73]. Compensatory autophagy is induced in response to UPS impairment in the SBMA fly model in an HDAC6-dependent manner [73]. Furthermore, HDAC6 overexpression rescued degeneration associated with UPS dysfunction accelerating the rate of mutant AR clearance through autophagy [73].

 Other therapeutic approaches to augment autophagy induction are the use of rapamycin, an mTOR inhibitor [73], and trehalose [34, 74]. However, a recent report using knock-in SBMA mouse suggests that autophagy activators are unlikely to be effective therapeutics for the subset of protein aggregation disorders such as SBMA where nuclear localization of the mutant protein is required for toxicity [75]. This suggests the need for due care in the use of autophagy augmentation therapies.

## 5. Modulation of Mitochondrial Function

Mitochondrial dysfunction has been implicated in various neurodegenerative diseases, including Huntington's disease, amyotrophic lateral sclerosis and Friedreich's ataxia [76, 77]. Expression of the mutant AR in cell cultures results in depolarization of the mitochondrial membrane and an elevated level of reactive oxygen species, which is blocked by treatments with the antioxidants coenzyme Q10 and idebenone (Figure 1) [78]. Mitochondrial dysfunction in SBMA is caused either by the indirect effects on the transcriptional repression of nuclear-encoded mitochondrial genes such as the peroxisome proliferator-activated receptor-*γ*-coactivator 1 (PGC-1) (Figure 1) and the mitochondrial specific antioxidant superoxide dismutase 2 or through direct effects of the mutant protein on mitochondria or both [78]. Therapeutic interventions targeted for mitochondrial function, UPS, or autophagy system might be promising treatments for all types of polyglutamine diseases.

## 6. Conclusions

SBMA is the first identified member of nine polyglutamine diseases [5], and due to its advantages through the well-investigated disease-causing protein structure and function, it has a leading place among polyglutamine diseases, especially from the standpoint of development of disease-modifying treatment as represented by hormonal therapies. However, despite dramatic efficacy in animal studies, hormonal therapies are not successfully translated into the clinical field at present [37, 38]. In consideration of the limited availability of participants, together with the slow progression of symptoms, clinical trials of SBMA should be carefully designed in terms of endpoints, sample size, duration, and inclusion and exclusion criteria [79, 80]. Other than hormonal therapies, therapeutic strategies have been developed to target many steps of the exertion of mutant AR toxicity as described in this article. It is possible that together with hormonal therapy preventing nuclear translocation of mutant AR, additional therapies targeted for the molecular events occurring after this translocation will strengthen the therapeutic effect for SBMA.
